# Performance and economic efficiency analysis of an integrated, outdoor fan-ventilated cooling device

**DOI:** 10.1016/j.heliyon.2023.e13927

**Published:** 2023-02-21

**Authors:** Ja-Kang Yang, Hyun-Je Lee, Sun-Hyo Park, Young-Tae Chae, Jong-Su Choi, Doo-Yong Park

**Affiliations:** a Korea Research Institute of Mechanical Facilities Industry, South Korea; b Research Institute, WooWon M&E Inc, South Korea; c Sustainable Architecture Institute, South Korea; d Department of Architectural Engineering, Gachon University, South Korea; e Soloenc, South Korea; f Building Energy Research Center, KCL (Korea Conformity Laboratories), South Korea

**Keywords:** Cooling efficiency, Load reduction, Condenser, Energy consumption, EnergyPlus, Economic efficiency

## Abstract

Recently, the importance of mechanical facilities in charge of the safety and comfort of occupants in buildings has once again been highlighted in accordance with global social issues such as the spread of COVID-19. In response, various ventilation systems are being developed to improve indoor air quality, and efforts are being made to satisfy the indoor comfort of the occupants. Such advanced facilities allow occupants to secure indoor air quality, while frequent ventilation systems can affect the cooling and heating load in the building, and there is also a problem that it can occupy a relatively large amount of space in the building. This study proposes an integrated, outdoor fan-ventilated cooling device and analyzes its performance and economic efficiency. The EnergyPlus simulation program was used to model two types of systems for comparison: an existing (base) model with a condenser located in the outdoor unit, and a developed model with the condenser integrated within the cooling system. The state of the air passing through the condenser was analyzed prior to comparing the efficiency of the integrated, outdoor fan-ventilated cooling device, followed by an in-depth analysis of the performance and economic efficiency based on total energy consumption. In Case 1, the air passing through the cooling system was approximately 5 °C lower than the base model and showed 11% peak load reduction in comparison to the maximum energy consumption. Additionally, a comparison between regions with different outdoor air temperatures showed an average cost reduction of 16% in Daejeon and Busan City.

## 1 Introduction

Interest in managing indoor spaces where the modern population spend at least 80% of their time has increased following research on the negative effects of highly concentrated indoor pollutants on the health and productivity of indoor occupants [1,2]. Therefore, air purification technologies that withdraw indoor air pollutants such as fine dust and radon are in demand [3]. Ventilation is discussed as a necessity among experts for preventing airborne transmissions including SARS, MERS, and COVID-19 and is considered a crucial factor among the general population for maintaining sound indoor air quality [4].

Jeong et al. classified ventilation methods as natural, mechanical, or hybrid [5]. Natural ventilation is suitable for energy-efficient buildings independent of mechanical systems but affected by the outdoor climate [6,7]. Mechanical ventilation that consumes significant energy and spatial volume has been adopted in the majority of modern structures [8]. Mechanical ventilation systems account for <40% of structural energy sectors in Europe and the United States and 50% of heating, ventilation, and air conditioning (HVAC) systems [9].

Most domestic ventilation systems combine evaporative cooling in dedicated outdoor air systems and apply thermal energy reuse through heat exchangers [10]. Recently, research on performance improvement through the use of dehumidifiers has been initiated [10]. Research on ventilation and air purifier systems with filters that eliminate indoor air pollutants has been performed [11]. Research on mechanical ventilation systems is ongoing to facilitate indoor air discharge due to the recent energy conservation regulations and the increase in the use of high-performance, structural insulating material [12,13]. In Europe, ventilation norms, EN 13779 and EN 15251, for non-residential buildings and indoor air quality, respectively, have been developed to automatically optimize the necessary amount of ventilation in a given space. Technical research on thermal recovery from exhaust gas is in progress [14,15]. Efficient operation and maintenance of mechanical ventilation devices installed as a result of increases in global electricity prices is problematic due to their low utility [16]; research on control systems in construction is being is conducted to overcome this limitation and reduce energy consumption from HVAC systems [17].

Santos et al. applied thermal energy storage technology using phase change material (PCM) to an integrated mechanical ventilation system and to reduce the error of simulated results via onsite ventilation and air conditioning measurements [18]. The increase in air flow enhanced thermal comfort and indoor air quality, and indoor air temperature was maintained within the desired range. Cooling capacity was dependent on the volume of PCM. Increasing the cooling capacity required an increase in PCMs but increasing the installation area remained a challenge.

Jiying et al. quantified the local thermal comfort and ventilation effects near humans by performing computational fluid dynamics (CFD) simulations on a radiant floor cooling system (RFCS) and displacement ventilation (DV), and combined, ductless, personalized ventilation (DPV) [19]. The low-temperature air absorbed by the DPV system tended to increase the temperature near the person, and the absorbed air tended to be more consistent with a lower DPV absorption height. The thermal environment was agreeable when the DPV flow rate was 5 L s^−1^ and absorption height was 0.1 m. Combining DPV, RFCS, and DV demonstrated that the local thermal environment near the occupant and indoor air quality could be improved.

Pinar et al. studied the application of energy-efficient, evaporative cooling, from which a high efficiency rate and cost effectiveness was derived in high-temperature and dry climatic zones [20]. Subsequent research on integrating the evaporative cooling system and liquid and gaseous driers is ongoing to fulfill regional, climate-specific demands. It is expected that the temperature range and thermal comfort desired by the occupant can be provided under any climatic condition.

Wang et al. developed an integrated condensing, heat recovery device to analyze the energy consumption of the system and calculate the annual recovery in static and dynamic investments [21]. The operating principle of this system was based on the recovery of the exhaust gas energy by developing the condenser of the exhaust path and condensing mixed gas. This method was more efficient and environmentally friendly, and the coefficient of performance (COP) could be improved. Significant profitability and value were demonstrated as data analysis on the investment recovery period showed that the period for static and dynamic investment was 3.8 years and 4.7 years, respectively.

Bordignon et al. studied integrated systems that combined heat exchangers for heating and cooling residential structures [22]. The impact of the integrated system on user energy consumption represented a 2% increase in power consumption, but the structural energy supply increased by 8% in comparison to the system without a heat exchanger.

Erjian Chen et al. studied Absorption-compression hybrid systems that consist of absorption and vapor compression cycle have advantages on both energy cost-effectiveness [23]. In this study, they propose two operating modes, subcooling mode and cascade mode. The study of this paper can function as guidance for design of the air-cooled absorption-compression integrated air conditioning.

Gauray et al. a small-scale triple-hybrid air-conditioning system operated by biomass and solar energy resources is experimentally investigated [24]. In this study, a combined experimental and simulation analysis of a triple-hybrid grid-independent cooling system has been carried out for achieving the net-zero-energy criterion and to address various environmental concerns. The system and the results are concluded to offer a sustainable solution towards the stubble burning problem and crop residue management for small and medium-scale applications.

Gauray et al. thermally driven vapor absorption-based air-conditioning systems possess many advantages over the compression-based systems [25]. In this study, Assessment has been carried out on a small office building to access the energy saving potential and thermal performance of a triple-hybrid absorption-based building cooling system against a conventional compression-based system. Two cases are studied for the absorption system, the overall maximum energy saving potential of absorption system against conventional system can be achieved up to 34.1%. The maximum value of solar fraction obtained from the simulation study is around 98.4%.

Although many individual studies have been conducted on the efficiency of ventilation systems, cooling systems, and energy savings, studies on systems that combine them are insufficient. While such advanced arrangements improve indoor air quality, frequent application of ventilation systems may affect the cooling and heating load. Therefore, a study quantitatively analyzing the efficiency and energy savings of the system combined with the existing ventilation system and cooling system is essential and has new novelty.

In addition, there is an advantage in space utilization because an outdoor unit installation space is not required separately. This paper proposes an integrated, outdoor fan-ventilated device to address the abovementioned limitations and analyzes its performance and economic efficiency.

## 2 Experimental methods

### 2.1 Integrated, outdoor fan-ventilated cooling device

**Fig. 1:**
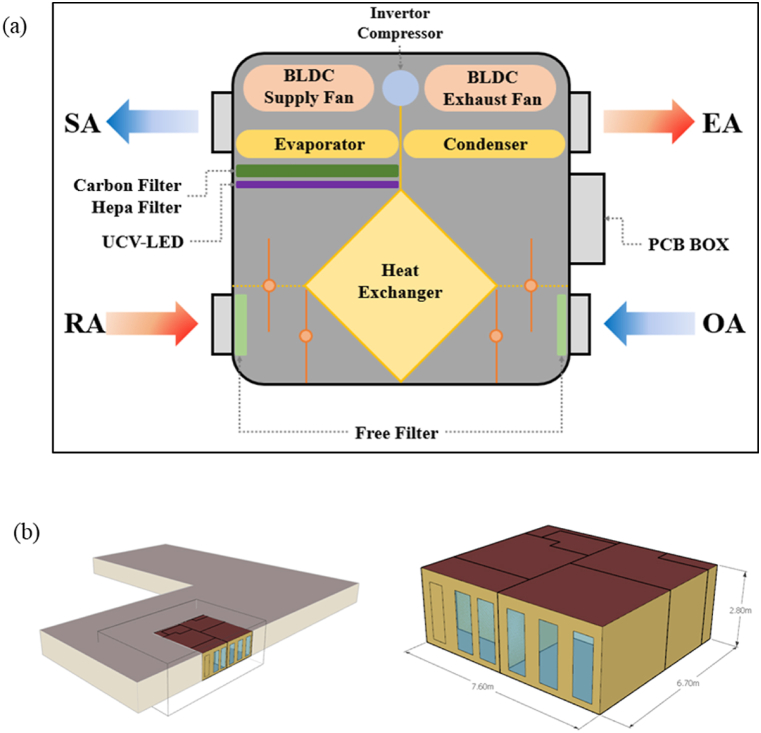
Configuration of integrated, total heat exchanger cooling system and structure of application ((a) System structure, (b) Building modeling).

In the existing system, heat recovery ventilation system, heat pump, and air purifier are installed separately in the building for cooling, ventilation, and air purification. The integrated, outdoor fan-ventilated cooling device proposed in this paper and its structure of application are shown in Fig. 1(a). The integrated ventilator is able to perform cooling, ventilation, air purification, and dehumidification. The outdoor fan is indistinct and internally combined. In comparison to non-inverter type systems, a relatively efficient and complex ventilation is possible through the application of the inverter compressor. A heat exchanger was included to minimize the cooling load during ventilation. The integrated system is more efficient than previous systems as installation is simple and the initial investment costs are low. During cooling, external air passes through the condenser, inverter compressor, and evaporator before being supplied internally. This paper examines the efficiency of the system by analyzing the state of the air passing through the condenser. In addition, there is an advantage in space utilization because an outdoor unit installation space is not required separately.

**Fig. 2:**
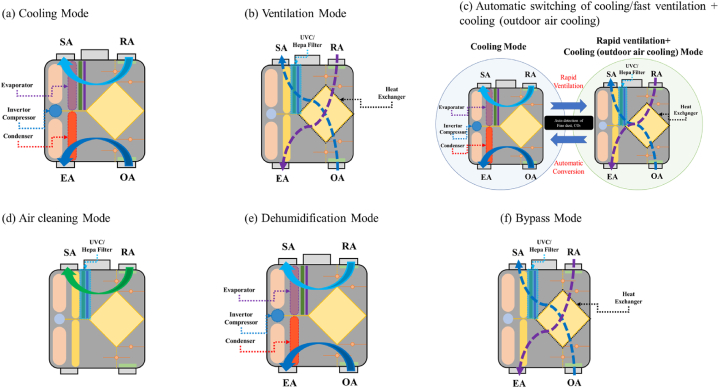
Operation mode of integrated, total heat exchanger cooling system ((a) Cooling mode, (b) Ventilation mode, (c)Rapid Ventilation cooling mode, (d)Air cleaning mode, (e)Dehumidification mode, (f)Bypass mode).

The operation mode of integrated, total heat exchanger cooling system is shown in Fig. 2(a)–(f). In the cooling mode (a), outdoor air passes through the condenser and the inverter compressor, and is mixed with indoor air in the evaporator to be cooled and supplied to the room. And, it is possible to ensure cooling capacity with minimum power consumption by applying an inverter compressor for the first time in complex ventilation systems, and electricity charges are reduced by about 35%.

In the ventilation mode (b), contaminated indoor air and fresh outdoor air are mixed in the heat exchanger and supplied to the room through the UVC/HEPA filter. Like a typical ventilation system, contaminated air in the room can be exchanged for fresh outside air.

The automatic switching mode of cooling/fast ventilation + cooling (outdoor air cooling) (c) automatically switches to rapid ventilation + cooling mode by detecting indoor fine dust and carbon dioxide concentration during cooling operation, and automatically switches to cooling mode when indoor air pollution becomes comfortable. The amount of ventilation through the typical ventilation system is 0.5 times/h, but if this mode is operated, ventilation is possible 4 to 5 times/h, and the effect of ventilating by opening the window can be obtained. It is possible to quickly remove indoor pollutants in a shorter time than in a typical ventilation mode and recover them to a clean state.

The air cleaning mode (d) automatically adjusts the air volume according to the indoor fine dust and carbon dioxide concentration and resupplies it indoors through a UV/HEPA filter. It is possible to clean the air volume of 300CMH or more, and it is possible to automatically adjust the air volume by detecting indoor pollutants.

In the dehumidification mode (e), external air passes through the condenser and the inverter compressor. And is mixed with indoor air in the evaporator, and the dehumidified air is supplied to the room. It is possible to dehumidify by automatically adjusting the air volume according to the user’s set humidity, and there is an advantage that indoor premise dehumidification is possible with one device.

The bypass mode (f) is a mode that enables ventilation with fresh air without heat exchange on a day when the temperature difference between indoor and outdoor is small, and prevents intersection of supply and discharge.

### 2.2 Subject area

A dynamic simulation program, EnergyPlus, was used to analyze the performance of the integrated, outdoor fan-ventilated cooling device. By considering the capacity of the developed cooling system and applying weather data, Incheon City, a small-scale residential structure with an actual operating area of 33 m^2^, was chosen as the subject. The modeling image of the unit to be analyzed is shown in Fig. 1(b). A single room as a discrete unit, which was located at the center of the structure, was analyzed. The room was sub-divided into two sections. While the condenser in the conventional model was placed in the outdoor fan room, the developed model integrated the condenser into the cooling system. The state of the air passing the condenser was analyzed prior to evaluating the efficiency of the integrated, outdoor fan-ventilated cooling device. Based on total energy consumption, an in-depth analysis of the performance and economic efficiency was conducted.

### 2.3 Engineering analysis

#### 2.3.1 Simulation settings

In EnergyPlus, the energy consumed by the compressor and condenser fan in the air conditioner package was calculated as:

(1) *Power* = *(Q*_*total*_*)(EIR)(RTF)*

Here, Q_total_ was calculated (Eq. (2)) and the Energy Input Ratio (EIR) was obtained by the load factor of the air flux over the total load (Eq. (3)). The energy input ratio factor (EIRTempModFrac) according to the air temperature near the evaporator, energy input ratio factor (EIRFlowModFrac) dependent on the evaporator air flux, and the coefficient of performance (COP) during rated operation were used to calculate the EIR.

(2) *Q*_*total*_ = *(Q*_*total, rated*_*)(TotCapTempModFac)(TotCapFlowModFac*)

(3) $$\mathrm{EIR}=\mathrm{Energy}\;\mathrm{input}\;\mathrm{ratio}=\frac{1}{{\mathrm{COP}}_{\mathrm{rated}}}\left(\mathrm{EIRTempModFac}\right)\left(\mathrm{EIRFlowModFac}\right)$$

The run time fraction (RTF) of Eq. (1), based on the operation of the system, can be derived by Eq. (4). The part load ratio (PLR) was obtained using Eq. (5), and the PartLoadFrac, representing the decrease in efficiency according to the fluctuations in compressor cycle associated with the PLR, was derived from the polynomial regression in Eq. (6).

(4) $$RTF=\frac{PLR}{PartLoadFrac}$$

(5) $$PLR=Part-Load\;Ratio=\frac{Sensible\;Cooling\;Load}{Steady-state\;Sensible\;Cooling\;Capacity}$$

(6) *PartLoadFrac = a* + *b PLR + c PLR*^*2*^ + *d PLR*^*3*^

The constant in Eq. (6) varies according to the properties of the direct expansion coil. In the case of a linear relationship, *c* and *d* can be adjusted to 0.

If the total ton of refrigeration (RT) is determined by the above equation, the amount of heat released from the condenser can be obtained by Eq. (7).

(7) *Q*_*cond*_ *= Q*_*total*_*(1* + *EIR)*

A total heat exchanger and air-cooled, packaged air conditioner currently used in South Korea was used to process the hourly air conditioner energy consumption in summer from June 1 to September 31, 2021. Two types of system operation were used, an outside air condenser cooling, and condenser cooling using air near the total heat exchanger output. Standard meteorological data based on TMY/ISO 15927-4:2005 was used for the corresponding region (Table 1).

**Table 1:** Simulation input data to analyze the performance of an integrated, outdoor fan-ventilated cooling device.

Index	Base Model	Case 1
System configuration	DOAS package air conditioner	integrated ventilation, cooling device
Nominal Cooling Capacity [W]	12,500	
TotCapTempModFac	0.94 + 9.5E^−3^*x* + 6.8E^−4^*x*^2^ - 1.1E^−2−^*y* + 5.2E^−6^y^2^ - 9.7E^−6^*xy* (*x* > 12.78, *y* < 23.89)	
TotCapFlowModFac	0.34 + 3.4E^−2^*x* - 6.2E^−5^*x*^2^ + 4.9E^−3^*y* - 4.3E^−4^y^2^ - 7.2E-4*xy* (*x* > 18, *y* < 46.11)	
Condenser location	utility room	inside system
Condenser process air	utility room air	relief air of heat recovery
Condensor air flow rate [m^3^ s-^1^]	1.9	
Nominal COP [−]	3.0	
Weather data	Inchoen Int’l airport TMYx	
Run period	7/1 00:00–9/31 24:00 (92 d)	
System operation hours	08:00–19:00 (12 h)	
Indoor Set point Temperature [°C]	24	
Process air mass flow	1.0 m^3^ s^−1^	

COP, coefficient of performance; DOAS, dedicated outdoor air system; *TotCapTempModFrac, total cooling capacity function of temperature fraction*; *TotCapFlowModFrac, total cooling capacity function of flow fraction*.

## 3 Engineering analysis

### 3.1 Dynamic structural energy

#### 3.1.1 Condenser air properties

**Fig. 3:**
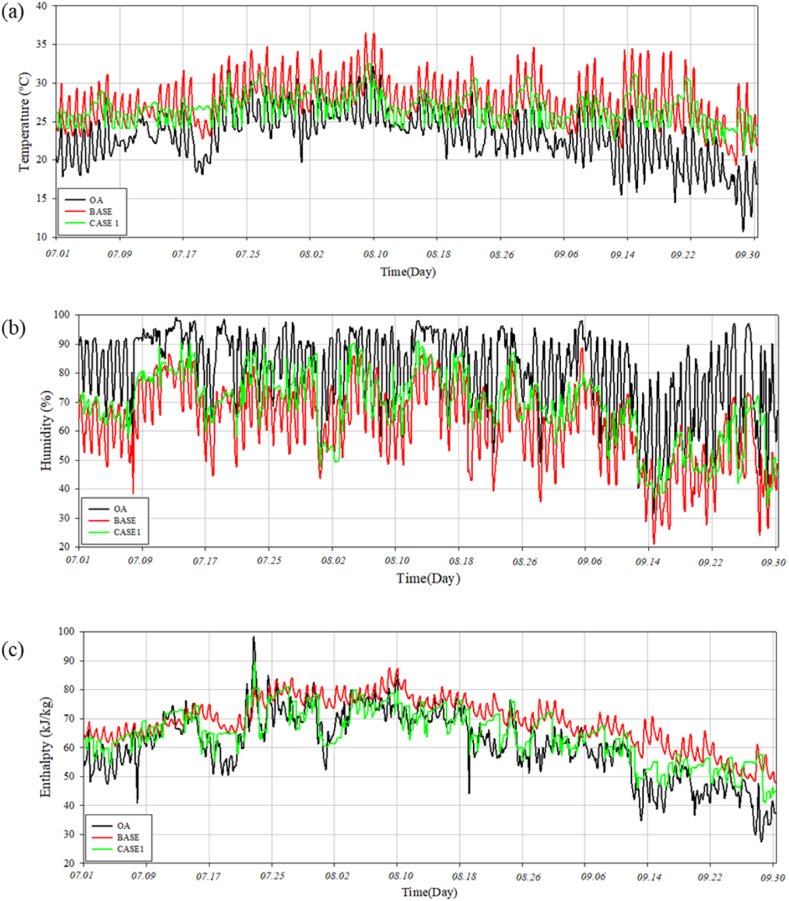
During the run period near the total heat exchanger exhaust during the analysis period (June 1–September 31, 2021) ((a)Hourly open-air temperature, (b)humidity, (c)enthalpy).

The temperature around the outdoor fan room, where the condenser was mounted, was ∼5 °C above the atmospheric temperature due to solar radiation and condenser heating Fig. 3(a)–(c). The temperature near the total heat exchanger exhaust and the indoor air temperature during operation were similar. High temperature and humidity during summer was well reflected, and the relative humidity altered rapidly according to the climate. While the relative open-air humidity reacted sensitively to the climate, the relative humidity of the outdoor fan room was maintained below that of the open air according to the rise in temperature in the outdoor fan room. During its operation, a 50–60% rate of relative humidity was maintained at the exhaust end of the total heat exchanger, which significantly altered the measured gas enthalpy.

#### 3.1.2 Operational characteristics of peak load during the day

**Fig. 4:**
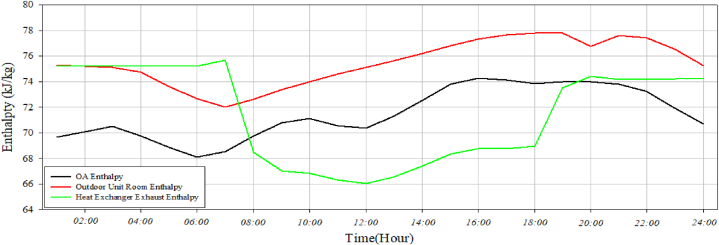
Hourly enthalpy variation of the day of peak load (12 August 2021).

Fig. 4 shows the hourly changes in enthalpy of the open air, internal air of the outdoor fan room, and exhaust gas of the total heat exchanger on August 12, during maximum open-air temperature. The relative humidity of the outdoor fan room was low. Thus, the enthalpy was measured lower than that of the open air but a similar pattern was exhibited. The enthalpy at the exhaust end of the total heat exchanger was able to be maintained constant at 66–69 kJ kg^−1^ during its operation.

**Fig. 5:**
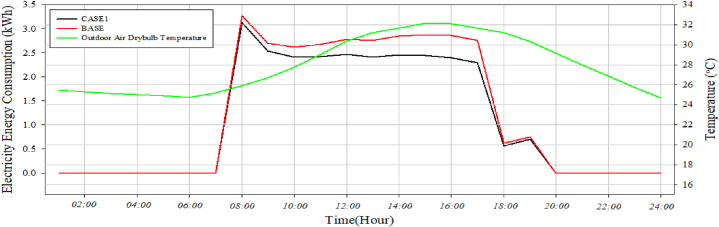
Hourly cooling system energy consumptions with outdoor air temperature (August 11, 2021).

Fig. 5 presents the hourly changes in outdoor air drybulb temperature and cooling system energy consumed by the compressor and condenser fan.

In Case 1, the maximum energy consumption was 3134.60 Wh at 08:00, which was the.

Onset of occupancy, demonstrating an 11% peak load reduction compared to the maximum energy consumption of 3270.81 Wh of the existing method, which utilizes the air within the outdoor fan room. This improvement in performance was attributed to enhanced condensation efficiency as the temperature and humidity of the exhaust gas from the total heat exchanger was similar to the air indoors rather than the outdoor fan room. Given the current system that is heavily determined by electricity bills, the difference in energy consumption between Case 1 and the Base Model was proportional to the duration of peak load. Therefore, it was determined that applying the developed model in commercial structures would be cost effective.

#### 3.1.3 Daily cooling energy consumption

**Fig. 6:**
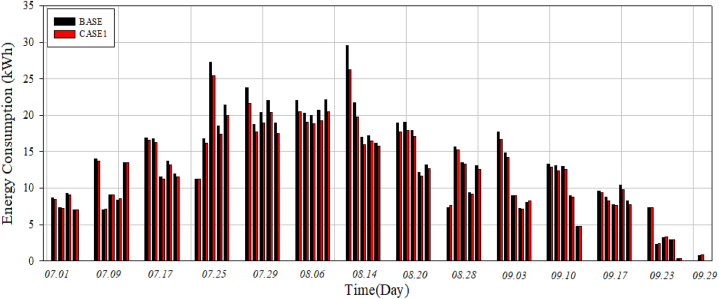
Comparison of the cooling system energy consumption of the developed and the conventional cooling models.

Fig. 6 compares the daily energy consumption of Case 1 and the Base Model over a total of 66 days of week operation. At peak load, a maximum of 11% cooling energy could be saved. The days exhibiting reversed enthalpy due to the temperature decrease in the outdoor fan room relative to the outdoor air temperature was negligible. Thus, the developed model was more energy efficient during cooling. The estimated rate of electricity cost reduction from monthly cooling energy consumption in Incheon City was 8.2% in August and 2.8% in September. The expected rate of electricity cost reduction from July to September was 5.6%. Table 2 compares the weather data from two additional cities, Daejeon and Busan, apart from the default data of Incheon City. In Daejeon, the reduction rate was 20% in July and August, 10% in September, and on average, 16%. In Busan, the maximum reduction rate in August was 20%, the minimum in September was 11%, and the average was 16%.

**Table 2:** Monthly cooling energy consumptions and utilities with the developed system.

Location	Month	Conventional	Developed	Utility savings []
		cooling energy consumption [kWh] (monthly cooling utility, )	cooling energy consumption [kWh] (monthly cooling utility, )	
Incheon	7	335.5056 (50,840)	321.3907 (47,890)	8090
	8	366.1808 (57,360)	344.9654 (52,730)	
	9	171.6348 (18,460)	166.5898 (18,130)	
Daejeon	7	419.4348 (77,010)	389.5906 (62,210)	32,020
	8	408.703 (73,540)	374.4444 (59,050)	
	9	229.6312 (28,510)	216.1207 (25,780)	
Busan	7	417.7351 (76,380)	391.3584 (62,630)	32,540
	8	411.6899 (74,480)	379.9495 (60,100)	
	9	299.7081 (43,250)	278.2066 (38,840)	

### 3.2 Summary

In Section 3.1, the results of dynamic structure energy analysis are graphically shown on the condensers air properties characteristics, peak load system operational characteristics, and daily cooling energy consumption.

Fig. 3(a)–(c) shows changes in air conditions such as outside air, outdoor air chamber, and outside air temperature, relative humidity, and enthalpy on the exhaust side of the heat transfer exchanger. For the air passing through the condenser, in the case of the outdoor unit room where the condenser is installed, the internal heat is analyzed to be higher than the outside temperature, and the exhaust temperature is similar to the indoor air at the operating time. The relative humidity was found to change rapidly due to the influence of weather on the relative humidity of the outside air.

In Fig. 4, Fig. 5, the enthalpy change and cooling energy consumption for Peak Load were shown, and it was analyzed that the outdoor unit room had a similar tendency although the enthalpy was lower than the outside air due to the low relative humidity. In Case 1, the maximum energy consumption was 3134.60 Wh at 8:00 a.m. when the re-real time starts, which is about 11% of the maximum energy consumption (3270.81 Wh) of the conventional method using the air in the outdoor unit room.

In Fig. 6, the daily energy consumption of Case 1 and Base Model was shown during daytime operation, and the maximum load day was analyzed to have a cooling energy saving effect of up to 11%.

## 4 Discussion

Previous literature on systems that combine ventilation, cooling, and heating analyzed efficiency and payback period based on recovering exhaust gas energy. In addition, in the case of a system that integrates cooling and heating and heat exchangers, literature on increasing energy consumption has also been reported.

This study compared the performance and economic efficiency of the Base Model, a commonly used cooling device that places the condenser in an outdoor fan room, and an integrated, outdoor fan ventilation cooling device system (Case 1).

The air passing the cooling system in Case 1 was ∼5 °C lower than in the outdoor fan room of the Base Model, representing an ∼11% peak load reduction rate based on the maximum energy consumption. This result was attributed to the improvement in condensation efficiency because the temperature and humidity of the total heat exchanger exhaust gas in Case 1 was closer to the indoor air temperature than the air in the outdoor fan room of the Base Model. A comparison of the daily energy consumption showed an 11% reduction in cooling energy during peak load, and the rates of energy cost reduction were on average 8.2% in August and 5.6% from July to September. In addition, a 16% cost reduction on average was examined in Daejeon and Busan, which had different outdoor air temperatures.

This study also has a limitation in that analysis was conducted only on small residential buildings according to the air volume and size of the developed combined system. In addition, in order to increase energy efficiency, it is necessary to analyze the energy saving rate by controlling strategy of cooling and heating period. In future studies, the research results will be generalized by comparing measurement data and simulation data for empirical buildings by control strategy and verifying energy savings.

When designing in the existing method, cooling facilities such as air conditioners were selected through cooling loads per area, and ventilation facilities were selected based on the standard of ventilation volume per person. On the other hand, when designing a combined system, there is no outdoor unit in the construction part, so it is possible to use the part advantageous for the space. In addition, cooling and ventilation can be performed through mode switching, and the cost reduction effect is excellent compared to each existing equipment. The results of this study are expected to be used for energy efficiency policies such as green remodeling and zero energy building certification systems.

## 5 Conclusion and recommendations

This study developed an integrated, outdoor fan-ventilated cooling device and analyzed its efficiency relative to a standard model. Positive results were obtained in overall energy efficiency. However, application is limited to the experimental results derived from a relatively small-scale, residential structural unit. Therefore, subsequent research on the operational methods associated with larger air conditioners is considered necessary for widespread application of the equipment.

## Author contribution statement

Ja-Kang Yang, Doo-Yong Park: Conceived and designed the experiments; Performed the experiments; Analyzed and interpreted the data; Contributed reagents, materials, analysis tools or data; Wrote the paper.

Hyun-Je Lee, Sun-Hyo Park, Young-Tae Chae, Jong-Soo Choi: Performed the experiments; Analyzed and interpreted the data; Contributed reagents, materials, analysis tools or data.

## Funding statement

This work was supported by Korea Institute of Planning and Evaluation for Technology in Food, Agriculture and Forestry (10.13039/501100014189IPET) and Korea Smart Farm R&D Foundation (KosFarm) through Smart Farm Innovation Technology Development Program, funded by Ministry of Agriculture, Food and Rural Affairs (10.13039/501100003624MAFRA) and Ministry of Science and ICT (10.13039/501100014188MSIT), Rural Development Administration (10.13039/501100003627RDA)(grant number. 421008-04-2-HD080). And also this work is supported by the 10.13039/501100007694Korea Agency for Infrastructure Technology Advancement (10.13039/501100007694KAIA) grant funded by the 10.13039/501100003565Ministry of Land, Infrastructure and Transport (grant number. 22PIYR-B153277-04).

## Data availability statement

Data included in article/supplementary material/referenced in article.

## Declaration of interest’s statement

The authors declare no conflict of interest.

## Nomenclature

BLDC: Brushless direct current motor
CFD: Computational fluid dynamics
COP: Coefficient of performance
${COP}_{rated}$: Coefficient of performance at rated conditions (W/W)
DPV: Ductless, personalized ventilation
DOAS: Dedicated outdoor air system
DV: Displacement ventilation
EA: Exhaust air
EIR: Energy Input Ratio
EIRTempModFac: Energy Input Ratio function of temperature curve evaluated at the test condition of wet-bulb temperature of air entering the cooling coil and dry-bulb temperature of air entering the air-cooled (outdoor) condenser (dimensionless)
EIRFlowModFac: Energy Input Ratio function of flow fraction curve evaluated at a flow fraction of 1.0 (dimensionless)
HVAC: Heating, Ventilation, and Air Conditioning
OA: Outdoor air
PCM: Phase change material
PLR: Part load ratio
$Q_{total}$: Standard ration cooling capacity (W)
$Q_{total,rated}$: Rated total cooling capacity (W)
RA: Return air
RFCS: Radiant floor cooling system
RT: Ton of refrigeration
RTF: Run time fraction
SA: Supply air
TotCapTempModFac: Total cooling capacity function of temperature curve evaluated at the test condition of wet-bulb temperature of air entering the cooling coil and dry-bulb temperature of air entering the air-cooled (outdoor) condenser (dimensionless)
TotCapFlowModFac: Total cooling capacity function of flow fraction curve evaluated at a flow fraction of 1.0 (dimensionless)
